# The relationship between micronutrient status, frailty, systemic inflammation, and clinical outcomes in patients admitted to hospital with COVID-19

**DOI:** 10.1186/s12967-023-04138-y

**Published:** 2023-04-28

**Authors:** Josh McGovern, John Wadsworth, Anthony Catchpole, Conor Richards, Donald C. McMillan, Tadhg Kelliher, Emma Goodall, Ellie Murray, Terry Melaugh, Shannon McPhillips, Kathryn Brice, Katie Barbour, Sophie Robinson, Peter Moffitt, Olivia Kemp, Dinesh Talwar, Donogh Maguire

**Affiliations:** 1 Academic Unit of Surgery, School of Medicine, University of Glasgow, Level 2, New Lister Building, Glasgow Royal Infirmary, Glasgow, G31 2ER UK; 2 Clinical Biochemistry Department, Glasgow Royal Infirmary, Glasgow, G4 0SF UK; 3 Emergency Department, Glasgow Royal Infirmary, Glasgow, G4 0SF UK

## Abstract

**Background:**

Micronutrients have been associated with disease severity and poorer clinical outcomes in patients with COVID-19. However, there is a paucity of studies examining if the relationship with micronutrient status and clinical outcomes is independent of recognised prognostic factors, specifically frailty and the systemic inflammatory response (SIR). The aim of the present study was to examine the relationship between micronutrient status, frailty, systemic inflammation, and clinical outcomes in patients admitted with COVID-19.

**Methods:**

Retrospective analysis of prospectively collected data was performed on patients with confirmed COVID-19, admitted to hospital between the 1st April 2020–6th July 2020. Clinicopathological characteristics, frailty assessment, biochemical and micronutrient laboratory results were recorded. Frailty status was determined using the Clinical Frailty scale. SIR was determined using serum CRP. Clinical outcomes of interest were oxygen requirement, ITU admission and 30-day mortality. Categorical variables were analysed using chi-square test and binary logistics regression analysis. Continuous variables were analysed using the Mann–Whitney U or Kruskal Wallis tests.

**Results:**

281 patients were included. 55% (n = 155) were aged ≥ 70 years and 39% (n = 109) were male. 49% (n = 138) of patients were frail (CFS > 3). 86% (n = 242) of patients had a serum CRP > 10 mg/L. On univariate analysis, frailty was significantly associated with thirty-day mortality (p < 0.001). On univariate analysis, serum CRP was found to be significantly associated with an oxygen requirement on admission in non-frail patients (p = 0.004). Over a third (36%) of non-frail patients had a low vitamin B1, despite having normal reference range values of red cell B2, B6 and selenium. Furthermore, serum CRP was found to be significantly associated with a lower median red cell vitamin B1 (p = 0.029).

**Conclusion:**

Vitamin B1 stores may be depleted in COVID-19 patients experiencing a significant SIR and providing rationale for thiamine supplementation. Further longitudinal studies are warranted to delineate the trend in thiamine status following COVID-19.

## Introduction

Micronutrients, broadly classified into vitamins and trace elements, are a cornerstone to human metabolism [1]. From the maintenance of normal tissue function, to the prevention of disease, the role of micronutrients have been widely studied [2]. Specifically, in the normal functioning of the healthy immune system and regulation of oxidative stress [3–5]. It has been postulated that deficiencies in certain micronutrients can compromise the body’s innate and adaptive immune responses to pathogens [6]. Furthermore, that supplementation of micronutrients may improve clinical outcomes in critically ill patients admitted with sepsis [7].

Trace elements including copper, magnesium, selenium and zinc have been associated with disease severity and poorer clinical outcomes in patients with COVID-19 [8–11]. This had led to a corresponding call for supplementation of certain micronutrients to improve outcomes in patients with COVID-19 [12]. However, studies within the present literature have often been carried out using serum or plasma measurements that are recognised to be perturbed by the systemic inflammatory response [13]. This is likely to be a confounding factor given that an exaggerated host inflammatory response (the cytokine storm), is now recognised as a hallmark of severe COVID-19 [14–16]. Furthermore, results may have been confounded by micronutrient deficiencies being more prevalent in frail patients [17, 18], another robust prognostic factor in COVID-19 [19]. Therefore, it remains unclear whether there is a causal effect between micronutrient deficiencies and clinical outcomes in patients with COVID-19 [20].

In contrast to serum/plasma measurements, red-cell micronutrient concentrations are thought to be reliable markers of long-term status, not confounded by the systemic inflammatory response (SIR) [21, 22]. Such measures may be more useful markers for examining micronutrient status in patients with an acute inflammatory pathology such as COVID-19 [22]. While it has been postulated that deficiencies in vitamins, specifically B and D, may be rational therapeutic targets in patients with COVID-19, there is a paucity of studies examining the relationship with recognised prognostic factors, specifically frailty and SIR [23, 24]. Furthermore, within the current literature, studies examining the potential therapeutic benefit of micronutrient supplementation on clinical outcomes have often not reported baseline micronutrient values [25, 26]. As such, it remains unclear whether such deficiencies are a result of COVID-19 per se, or simply that COVID-19 occurs on a background of micronutrient deficiency. Therefore, the aim of the present study was to examine the relationship between micronutrient status, frailty, systemic inflammation and clinical outcomes in patients admitted with COVID-19. Firstly, to examine if there are micronutrient perturbations in patients admitted with COVID-19. Secondly, examine if micronutrient perturbations are independent of frailty status and the systemic inflammatory response in patients with COVID-19.

## Methods

A retrospective analysis was carried out of prospectively collected data on patients who presented to Glasgow Royal Infirmary or the Queen Elizabeth University Hospital, Glasgow, U.K., between the 1st April 2020–6th July 2020. In line with NHS policy, this study was approved by the NHS Greater Glasgow and Clyde biorepository research ethics committee. The study protocol (GN20AE307) was approved by the Northwest England—Preston research ethics committee (20/NW/0336) and registered with clinicaltrials.gov (NCT04484545), as previously described [27].

Patients with either a positive polymerase chain reaction (PCR) test or radiological changes characteristic of COVID-19 infection, reported on chest X-ray (CXR) or CT thorax, by a board-certified radiologist on admission were assessed for inclusion in the study. Patients who had samples taken for storage in the Biorepository at the time of presentation and subsequently analyzed for micronutrient status were included. Exclusion criteria were the absence of a recorded diagnosis of COVID-19 test result or assessment of any micronutrient measurement.

Routine demographic details, clinicopathological characteristics, frailty and nutritional assessments, as well as biochemical laboratory results were recorded. Age, sex, documented micronutrient assessment and diagnostic modality confirming COVID-19 infection, as well as date of admission and 30-day mortality status were minimal inclusion criteria. Age categories were grouped to </≥ 70 years. BMI was categorised as < 25/≥ 25 kg/m^2^. Co-morbidity data collected included a diagnosis of hypertension, heart failure, chronic obstructive pulmonary disease, type 2 diabetes mellitus, liver disease, chronic kidney disease and active cancer. Frailty was assessed using the 9-category Clinical Frailty Scale (CFS) [28]. Malnutrition was screened using the five-step Malnutrition Universal Screening Tool (MUST) [29]. Both frailty and MUST scores were identified from admission nursing assessments. Patients with CFS > 3 were categorized as frail. Patients were classified as no risk (MUST = 0), or at risk of malnutrition (MUST ≥ 1). Magnitude of SIR was determined using admission serum C-reactive protein (CRP). Values were categorised as ≤ 10/11–80/> 80 mg/L, as per previous studies of the relationship between micronutrient concentrations and SIR [13]. Admission serum albumin concentration values were categorised as ≥ 35/< 35 g/L. An autoanalyzer was used to measure serum CRP (mg/L) and albumin (g/L) concentrations (Alinity, Abbott Diagnostics, Abbott Park, Chicago, United States).

### Micronutrient analysis

Venous blood samples of approximately 5mls, collected from patients on admission to hospital in EDTA and non-gel heparin tubes were used for the measurement of micronutrients. Samples were centrifuged (3500×*g* for 15 min at 4 °C) and plasma was removed. Then, packed red cells were carefully prepared removing all remaining plasma and buffy coat prior to storage at − 70 °C. Samples were analysed within 6 months of collection. Samples from individual patients were assayed in the same batch to minimize analytical variation. In total, eight micronutrients (vitamins and trace elements) were measured in plasma or erythrocytes. Analysis was performed by the Scottish Trace Element and Micronutrient Diagnostic and Research Laboratory, a national accredited service.

Vitamin B1 (thiamine diphosphate; TDP) is present almost exclusively in red cells and so vitamin B1 status was assessed by measuring TDP in red cells. An HPLC system with post-column ferricyanide derivatization and fluorometric detection was used as described previously [30]. The TDP concentration in red cell was expressed as a haemoglobin (Hb) ratio in the sample (ng TDP/g Hb). The within-batch imprecision was 5.1% at 380 ng/g Hb.

Vitamin B2 (flavin adenine dinucleotide; FAD) measurement in whole blood and erythrocytes was based on the method of Speek and co-workers. Briefly, diluted red cell hemolysate were precipitated with 70% perchloric acid and centrifuged. Then, supernatant was injected for HPLC analysis. FAD was separated on an isocratic HPLC system with a reversed-phase C18 column and fluorescence detection. The within-batch imprecision for whole blood FAD was 4.8% at 384 nmol/L and 4.8% at 2.8 nmol/g Hb red cell FAD.

Vitamin B6 (pyridoxyl phosphate; PLP) concentrations in red cells were measured by HPLC using pre-column semi-carbazide derivatization and fluorescent detection as described previously [31]. The within-batch imprecision was 5.2% at 367 pmol/g Hb.

PLP, FAD and TDP concentrations in red cells were adjusted to haemoglobin (Hb) rather than to the volume of packed red cells because accurate pipetting of packed red cells is difficult due to the high viscosity. The HPLC system for measurement of vitamins B1, B2 and B6 consisted of a Waters solvent delivery system and a Waters fluorimeter, Model 2475 (Waters, Wilmslow, UK).

Inductively coupled plasma mass spectrometry (Agilent Technologies, Cheadle, UK) was used to measure plasma copper and selenium, as well as red cell selenium and iron. Plasma samples were diluted 1 in 20 with a 2% ammonia solution containing 50 µg/L germanium, which was present as an internal standard. Copper and selenium were measured in plasma using a 7900 inductively-coupled plasma mass spectrometer (Agilent Technologies, Inc., Santa Clara, CA, USA) operating in helium mode. The coefficient of variation for plasma selenium was < 4%.

Packed erythrocytes were diluted 1 in 80 with a 0.5% ammonia solution containing 50 µg/L scandium, which was present as an internal standard. Selenium and iron were a measured simultaneously in erythrocytes using a 7900 inductively-coupled plasma mass spectrometer operating in hydrogen mode for selenium and helium mode for iron. Erythrocyte selenium was expressed as a ratio to haemoglobin concentration to correct for potential inaccuracies associated with pipetting packed erythrocytes and minimise imprecision. Unlike plasma, erythrocytes are viscous making accurate pipetting difficult. Iron was measured as a surrogate for haemoglobin, the concentration of which was calculated using the following equation (where 64,456 is the molecular weight of haemoglobin in g/mol and the denominator is the number of atoms of iron per haemoglobin molecule):

$$ {\text{Haemoglobin}}\;\left( {{\text{g}}/{\text{L}}} \right) = {{\left( {{\text{Iron}}\;\left( {{\text{mol}}/{\text{L}}} \right) \times 64,456} \right)} \mathord{\left/ {\vphantom {{\left( {{\text{Iron}}\;\left( {{\text{mol}}/{\text{L}}} \right) \times 64,456} \right)} 4}} \right. \kern-0pt} 4}. $$

The coefficient of variation for erythrocyte selenium:haemoglobin ratio was < 6%.

Vitamin D, vitamin B12, magnesium and LDH were measured in serum by routine laboratory procedures using automated analysers (Alinity, Abbott Diagnostics). The CV for these methods was < 5%.

For all methods, Quality Assurance and Quality Controls were assessed using Certified Reference Materials and thorough external Quality Assessment schemes (data available on request).

### Statistical analysis

Demographic data, clinicopathological variables, CFS, BMI, MUST score, micronutrient level, CRP, albumin, oxygen requirement, ITU admission and 30-day mortality were presented as categorical variables. Categorical variables were analysed using chi-square test for linear-by-linear association. For categorical variables, Fisher’s exact test was used when value of single cell of a two-by-two table was n ≤ 5. Micronutrient concentrations were also presented as continuous variables and analysed using the Mann–Whitney U or Kruskal Wallis tests.

The relationships between clinicopathological variables were also examined using univariate and multivariate binary logistic regression with backward conditional method. Covariates with a significance value of p < 0.1 in the univariate analysis were included in the multivariate analysis.

The present study was testing the hypothesis that patients with COVID-19 were similar to other patient groups with regard to micronutrient status. Therefore, given the present analysis was exploratory in nature and no formal power calculation was carried out.

Missing data were excluded from analysis on a variable-by-variable basis. Two-tailed *p* values < 0.05 were considered statistically significant. Statistical analysis was performed using SPSS software version 25.0. (SPSS Inc., Chicago, IL, USA).

## Results

Of the 599 patients admitted to hospital during the study period, 281 patients underwent micronutrient analysis. The clinicopathological characteristics at presentation are shown in Table 1. 55% (n = 155) were aged ≥ 70 years old. 39% (n = 109) participants were male. 37% (n = 105) patients were ex-/current smokers and 10% (n = 27) had a history of alcohol excess. 50% (n = 140) patients had a history of hypertension, 31% (n = 87) a history of type 2 diabetes mellitus and 22% (n = 63) a history of chronic renal failure. 49% (n = 138) patients were categorised as being frail (CFS > 3). Of the 255 patients with a MUST recorded on admission, 16% (n = 40) were at risk of malnutrition (MUST ≥ 1). Of the 229 patients with a recorded BMI on admission, 62% (n = 143) had a BMI ≥ 25 kg/m^2^. 86% (n = 242) patients had a serum CRP > 10 mg/L. 67% (n = 189) patients had a serum albumin < 35 g/L. 28% (n = 78) of patients had an oxygen requirement on admission. 10% (n = 27) of patients were admitted to ITU. 27% (n = 77) patients died within 30 days of admission.

**Table 1:** Patient characteristics (n = 281)

Demographics	Frequency, n (%)
Age (years)	
< 70	126 (45%)
≥ 70	155 (55%)
Sex	
Male	172 (61%)
Female	109 (39%)
Smoking Hx	
Never	176 (63%)
Ex/current	105 (37%)
Alcohol excess Hx	
No	254 (90%)
Yes	27 (10%)
Hypertension	
No	141 (50%)
Yes	140 (50%)
Heart failure	
No	235 (84%)
Yes	46 (16%)
T2DM	
No	194 (69%)
Yes	87 (31%)
COPD	
No	238 (85%)
Yes	43 (15%)
Chronic renal failure	
No	218 (78%)
Yes	63 (22%)
Liver disease	
No	254 (90%)
Yes	27 (10%)
Active cancer	
No	252 (90%)
Yes	29 (10%)
Frail (CFS > 3)	
No	143 (51%)
Yes	138 (49%)
BMI (kg/m^2^, n = 229)	
< 25	86 (38%)
≥ 25	143 (62%)
MUST (n = 255)	
Low	215 (84%)
Medium–high risk	40 (16%)
Serum LDH (U/L, n = 264)	
Median (IQR)	218 (165–302)
≤ 240	156 (59%)
> 240	108 (41%)
Serum CRP (mg/L)	
Median (IQR)	58 (22–127)
≤ 10	39 (14%)
Nov-80	127 (45%)
> 80	115 (41%)
Serum albumin (g/L)	
Median (IQR)	32 (29–36)
≥ 35	92 (33%)
< 35	189 (67%)
Oxygen requirement	
No	203 (72%)
Yes	78 (28%)
ITU admission	
No	254 (90%)
Yes	27 (10%)
30-day mortality	
No	206 (73%)
Yes	75 (27%)

The median value of each micronutrient analysed as part of the study and prevalence of patients with values outside of the references range of that micronutrient is shown in Table 2.

**Table 2:** Median micronutrient values and prevalence of those with values out with reference ranges in patients admitted with COVID-19 (n = 281)

Micronutrients	Threshold
Red cell vitamin B1 (n = 194)	
Median (IQR)	326 (261–427) ng/g of Hb
≥ 275 ng/g of Hb	135 (48%)
< 275 ng/g of Hb	59 (30%)
Red cell vitamin B2 (n = 64)	
Median (IQR)	2.01 (1.73–2.52) nmol/g of Hb
≥ 1.0 nmol/g of Hb	64 (100%)
< 1.0 nmol/g of Hb	0 (0%)
Red cell vitamin B6 (n = 64)	
Median (IQR)	450 (362–586) pmol/g of Hb
≥ 250 pmol/g of Hb	62 (97%)
< 250 pmol/g of Hb	2 (3%)
Red cell selenium (n = 198)	
Median (IQR)	5.58 (4.85–6.46) nmol/g Hb
≥ 3.0 nmol/g Hb	198 (100%)
< 3.0 nmol/g Hb	0 (0%)
Plasma selenium (n = 262)	
Median (IQR)	0.76 (0.56–0.94) µmol/L
≥ 0.8 µmol/L	143 (55%)
< 0.8 µmol/L	119 (45%)
Serum vitamin B12 (n = 262)	
Median (IQR)	455 (292–819)
≥ 188 ng/L	251 (96%)
< 188 ng/L	11 (4%)
Serum vitamin D (n = 111)	
Median (IQR)	31 (19–71) ng/L
≥ 50 nmol/L	41 (37%)
< 50 nmol/L	70 (63%)
Serum magnesium (n = 264)	
Median (IQR)	0.871 (0.73–0.88) mmol/L
≥ 0.75 mmol/L	194 (73%)
< 0.75 mmol/L	70 (27%)
Plasma copper (n = 262)	
Median (IQR)	20 (16–23) µmol/L
≤ 22 µmol/L	172 (66%)
> 22 µmol/L	90 (35%)

The relationship between frailty status and clinicopatholgical characteristics, BMI, MUST, micronutrient level, systemic inflammatory response and clinical outcomes in patients with COVID-19 is shown in Table 3. On univariate analysis, frailty was significantly associated with age (p < 0.001), smoking history (p = 0.020), heart failure (p < 0.001), T2DM (p < 0.001), COPD (p < 0.001), chronic renal failure (p < 0.001), active cancer (p = 0.002), BMI (p < 0.001), median red cell vitamin B1 (p < 0.001), median red cell vitamin B2 (p = 0.013), median red cell selenium (p = 0.004), median plasma selenium (p < 0.001), low plasma selenium (p < 0.001), median serum magnesium (p < 0.001), low serum magnesium (p < 0.001), median serum LDH (p < 0.001), serum LDH (p = 0.001), median serum albumin (p < 0.001), serum albumin (p < 0.001) and thirty-day mortality (p < 0.001).

**Table 3:** The relationship between frailty status and clinicopatholgical characteristics, BMI, MUST, micronutrient level, systemic inflammatory response and clinical outcomes in patients with COVID-19 (n = 281)

	Not frail (CFS ≤ 3, n = 143)	Frail (CFS > 3, n = 138)	P^1^ value
Age (years)			**< 0.001**
< 70	104 (73%)	22 (16%)	
≥ 70	39 (27%)	116 (84%)	
Sex			0.113
Male	94 (66%)	78 (56%)	
Female	49 (34%)	60 (44%)	
Smoking Hx			**0.02**
Never	99 (69%)	77 (56%)	
Ex/current	44 (31%)	61 (44%)	
Alcohol excess Hx			0.481
No	131 (92%)	123 (89%)	
Yes	12 (8%)	15 (11%)	
Hypertension			0.211
No	77 (54%)	64 (46%)	
Yes	66 (46%)	74 (54%)	
Heart failure			**< 0.001**
No	130 (91%)	105 (76%)	
Yes	13 (9%)	33 (24%)	
T2DM			**< 0.001**
No	113 (79%)	81 (59%)	
Yes	30 (21%)	57 (41%)	
COPD			**< 0.001**
No	133 (93%)	105 (76%)	
Yes	10 (7%)	33 (24%)	
Chronic renal failure			**< 0.001**
No	128 (89%)	90 (65%)	
Yes	15 (11%)	48 (35%)	
Liver disease			0.13
No	133 (93%)	121 (88%)	
Yes	10 (7%)	17 (12%)	
Active cancer			**0.002**
No	136 (95%)	116 (84%)	
Yes	7 (5%)	22 (16%)	
BMI (kg/m^2^, n = 229)			**< 0.001**
< 25	27 (23%)	59 (53%)	
≥ 25	90 (77%)	53 (47%)	
MUST (n = 255)			0.071
Low	114 (88%)	101 (80%)	
Medium–high risk	15 (12%)	25 (20%)	
Median red cell vitamin B1 (ng/g Hb, n = 194)	304 (243–389)	362 (277–490)	**< 0.001**
Low red cell vitamin B1 (ng/g Hb, n = 194)			0.071
No	68 (64%)	67 (76%)	
Yes	38 (36%)	21 (24%)	
Median red cell vitamin B2 (nmol/g of Hb, n = 64)	1.93 (1.70–2.12)	2.32 (1.90–2.78)	**0.013**
Low red cell vitamin B2 (nmol/g of Hb, n = 64)			1
No	36 (100%)	28 (100%)	
Yes	0 (0%)	0 (0%)	
Median red cell vitamin B6 (pmol/g of Hb, n = 64)	443 (362–549)	478 (352–608)	0.771
Low red cell vitamin B6 (pmol/g of Hb, n = 64)			0.188
No	36 (100%)	26 (93%)	
Yes	0 (0%)	2 (7%)	
Median red cell selenium (nmol/g Hb, n = 198)	5.84 (5.02–6.93)	5.44 (4.65–6.04)	**0.004**
Low red cell selenium (nmol/g Hb, n = 198)			1
No	108 (100%)	90 (100%)	
Yes	0 (0%)	0 (%)	
Median plasma selenium (µmol/L, n = 262)	0.86 (0.59–1.00)	0.66 (0.54–0.84)	**< 0.001**
Low plasma selenium (µmol/L, n = 262)			**< 0.001**
No	78 (59%)	41 (31%)	
Yes	54 (41%)	89 (69%)	
Median serum vitamin B12 (ng/L, n = 262)	496 (297–862)	448 (290–790)	0.307
Low serum vitamin B12 (ng/L, n = 262)			1
No	126 (96%)	125 (95%)	
Yes	5 (4%)	6 (5%)	
Median serum vitamin D (nmol/L, n = 111)	32 (19–62)	27 (16–77)	0.813
Low serum vitamin D (nmol/L, n = 111)			0.427
No	15 (33%)	26 (40%)	
Yes	31 (67%)	39 (60%)	
Median serum magnesium (mmol/L, n = 264)	0.83 (0.77–0.90)	0.78 (0.70–0.85)	**< 0.001**
Low serum magnesium (mmol/L, n = 264)			**< 0.001**
No	109 (83%)	85 (64%)	
Yes	23 (17%)	47 (36%)	
Median plasma copper (µmol/L, n = 262)	20 (16–24)	20 (17–23)	0.391
High plasma copper (µmol/L, n = 262)			0.226
No	82 (62%)	90 (69%)	
Yes	50 (38%)	40 (31%)	
Median (IQR) serum LDH (U/L, n = 264)	234 (177–341)	193 (160–252)	**< 0.001**
Serum LDH (U/L, n = 264)			**0.001**
≤ 240	68 (51%)	42 (32%)	
> 240	64 (49%)	90 (68%)	
Median (IQR) serum CRP (mg/L)	69 (24–146)	51 (17–109)	0.072
Serum CRP (mg/L)			0.05
≤ 10	16 (11%)	23 (17%)	
Nov-80	61 (43%)	66 (48%)	
> 80	66 (46%)	49 (35%)	
Median (IQR) serum albumin (g/L)	33 (30–37)	31 (27–34)	**< 0.001**
Serum albumin (g/L)			**< 0.001**
≥ 35	61 (43%)	31 (23%)	
< 35	82 (57%)	107 (77%)	
Oxygen requirement			0.093
No	97 (68%)	106 (77%)	
Yes	46 (32%)	32 (23%)	
ITU admission			0.187
No	126 (88%)	128 (93%)	
Yes	17 (12%)	10 (7%)	
30-day mortality			**< 0.001**
No	125 (87%)	79 (57%)	
Yes	18 (13%)	59 (43%)	

Significant p values are shown in bold

^1^p value is from χ^2^ analysis or Fisher’s exact test when value of single cell n ≤ 5 for categorical variables and Mann–Whitney U test for continuous variables

The relationship between clinicopatholgical characteristics, BMI, MUST, micronutrient level, systemic inflammatory response and clinical outcomes in patients with COVID-19 who were not frail, stratified by CRP, is shown in Table 4. On univariate analysis, serum CRP was found to be significantly associated with median red cell vitamin B1 (p = 0.029), median red cell vitamin B6 (p = 0.016), median serum magnesium (p < 0.001), low serum magnesium (p = 0.015), median plasma copper (p = 0.006), high plasma copper (p = 0.010), median serum LDH (p < 0.001), serum LDH (p = 0.001), median serum albumin (p < 0.001), serum albumin (p < 0.001) and oxygen requirement (p = 0.004).

**Table 4:** The relationship between clinicopatholgical characteristics, BMI, MUST, micronutrient level, systemic inflammatory response and clinical outcomes in patients with COVID-19 who were not frail, stratified by CRP (n = 143)

	CRP ≤ 10 (mg/L, n = 16)	CRP 11–80 (mg/L, n = 61)	CRP > 80 (mg/L, n = 66)	P^1^ value
Age (years)				0.649
< 70	13 (81%)	40 (66%)	51 (77%)	
≥ 70	3 (19%)	21 (34%)	15 (23%)	
Sex				0.062
Male	9 (56%)	36 (59%)	49 (74%)	
Female	7 (44%)	25 (41%)	17 (26%)	
Smoking Hx				0.331
Never	14 (87%)	40 (66%)	45 (68%)	
Ex/current	2 (13%)	21 (34%)	21 (32%)	
Alcohol excess Hx				0.93
No	15 (94%)	55 (90%)	61 (92%)	
Yes	1 (6%)	6 (10%)	5 (8%)	
Hypertension				0.632
No	9 (56%)	34 (56%)	34 (51%)	
Yes	7 (44%)	27 (44%)	32 (49%)	
Heart failure				0.05
No	12 (75%)	56 (92%)	62 (94%)	
Yes	4 (25%)	5 (8%)	4 (6%)	
T2DM				0.881
No	14 (87%)	45 (74%)	54 (82%)	
Yes	2 (13%)	16 (26%)	12 (18%)	
COPD				0.809
No	15 (94%)	56 (92%)	62 (94%)	
Yes	1 (6%)	5 (8%)	4 (6%)	
Chronic renal failure				0.086
No	14 (87%)	51 (84%)	63 (95%)	
Yes	2 (13%)	10 (16%)	3 (5%)	
Liver disease				0.467
No	14 (87%)	57 (93%)	62 (94%)	
Yes	2 (13%)	4 (7%)	4 (6%)	
Active cancer				0.405
No	14 (87%)	59 (97%)	63 (95%)	
Yes	2 (13%)	2 (3%)	3 (5%)	
BMI (kg/m^2^, n = 117)				0.161
< 25	6 (46%)	10 (20%)	11 (21%)	
≥ 25	7 (54%)	41 (80%)	42 (79%)	
MUST (n = 129)				0.939
Low	12 (92%)	46 (87%)	56 (89%)	
Medium–high risk	1 (8%)	7 (13%)	7 (11%)	
Median red cell B1 (ng/g Hb, n = 106)	336 (251–415)	333 (269–436)	289 (218–338)	**0.029**
Low red cell B1 (ng/g Hb, n = 106)				0.268
No	10 (71%)	30 (68%)	28 (58%)	
Yes	4 (29%)	14 (32%)	20 (42%)	
Median red cell B2 (nmol/g of Hb, n = 36)	2.02 (1.70–3.06)	1.73 (1.39–2.00)	2.02 (1.75–2.27)	0.085
Low red cell B2 (nmol/g of Hb, n = 36)				1
No	8 (100%)	15 (100%)	13 (100%)	
Yes	0 (0%)	0 (0%)	0 (0%)	
Median red cell B6 (pmol/g of Hb, n = 36)	642 (458–1288)	387 (312–495)	422 (368–562)	**0.016**
Low red cell B6 (pmol/g of Hb, n = 36)				1
No	8 (100%)	15 (100%)	13 (100%)	
Yes	0 (0%)	0 (0%)	0 (0%)	
Median red cell selenium (nmol/g Hb, n = 108)	6.64 (5.84–7.42)	5.63 (4.88–6.46)	6.10 (5.18–6.99)	0.055
Low red cell selenium (nmol/g Hb, n = 108)				1
No	14 (100%)	46 (100%)	48 (100%)	
Yes	0 (0%)	0 (0%)	0 (0%)	
Median plasma selenium (µmol/L, n = 132)	0.92 (0.46–1.00)	0.89 (0.60–1.06)	0.83 (0.59–0.96)	0.106
Low plasma selenium (µmol/L, n = 132)				0.925
No	8 (53%)	34 (63%)	36 (57%)	
Yes	7 (47%)	20 (37%)	27 (43%)	
Median serum vitamin B12 (ng/L, n = 131)	338 (253–453)	544 (282–846)	523 (361–1041)	0.895
Low serum vitamin B12 (ng/L, n = 131)				0.229
No	13 (87%)	53 (98%)	60 (97%)	
Yes	2 (13%)	1 (2%)	2 (3%)	
Median serum vitamin D (nmol/L, n = 46)	36 (15–73)	31 (19–63)	32 (21–65)	0.599
Low serum vitamin D (nmol/L, n = 46)				0.909
No	1 (20%)	9 (39%)	5 (28%)	
Yes	4 (80%)	14 (61%)	13 (72%)	
Median serum magnesium (mmol/L, n = 132)	0.80 (0.72–0.86)	0.80 (0.73–0.87)	0.87 (0.80–0.93)	**< 0.001**
Low serum magnesium (mmol/L, n = 132)				**0.015**
No	11 (73%)	41 (74%)	57 (92%)	
Yes	4 (27%)	14 (26%)	5 (8%)	
Median plasma copper (µmol/L, n = 132)	17 (13–22)	19 (16–23)	22 (19–25)	**0.006**
High plasma copper (µmol/L, n = 132)				**0.01**
No	12 (80%)	38 (70%)	32 (51%)	
Yes	3 (20%)	16 (30%)	31 (49%)	
Median (IQR) serum LDH (U/L, n = 132)	198 (133–283)	211 (164–269)	296 (220–379)	**< 0.001**
Serum LDH (U/L, n = 132)				**0.001**
≤ 240	5 (33%)	21 (38%)	42 (68%)	
> 240	10 (67%)	34 (62%)	20 (32%)	
Median (IQR) serum albumin (g/L)	38 (34–40)	36 (32–39)	32 (29–33)	**< 0.001**
Serum albumin (g/L)				**< 0.001**
≥ 35	12 (75%)	38 (62%)	11 (17%)	
< 35	4 (25%)	23 (38%)	55 (83%)	
Oxygen requirement				**0.004**
No	13 (81%)	48 (79%)	36 (54%)	
Yes	3 (19%)	13 (21%)	30 (46%)	
ITU admission				0.431
No	14 (87%)	56 (92%)	56 (85%)	
Yes	2 (13%)	5 (8%)	10 (15%)	
30-day mortality				0.166
No	15 (94%)	55 (90%)	55 (83%)	
Yes	1 (6%)	6 (10%)	11 (17%)	

Significant p values are shown in bold

^1^p value is from χ^2^ analysis for categorical variables or Kruskal–Wallis test for continuous variable

The relationship between low vitamin B1 and clinicopatholgical characteristics, BMI, MUST, systemic inflammatory response and clinical outcomes in non-frail patients admitted with COVID-19 is shown in Table 5. On univariate analysis, a low vitamin B1 was not significantly associated with age (p = 0.467), sex (p = 0.816), smoking Hx. (p = 0.968), alcohol excess Hx. (p = 0.679), BMI (p = 0.612), MUST (p = 0.253), serum CRP (p = 0.268), oxygen requirement (p = 0.224), ITU admission (p = 0.883), 30-day admission (p = 0.970).

**Table 5:** The relationship between low vitamin B1 and clinicopatholgical characteristics, BMI, MUST, systemic inflammatory response and clinical outcomes in non-frail patients admitted with COVID-19 (n = 106)

	Univariate analysis		Multivariate analysis	
	Odds ratio (95% confidence interval)	p-value	Odds ratio (95% confidence interval)	p-value
Age (< 70/ ≥ 70)	1.39 (0.58–3.33)	0.467	–	–
Sex (female/male)	1.11 (0.47–2.62)	0.816	–	–
Smoking Hx. (no/yes)	1.02 (0.44–2.35)	0.968	–	–
Alcohol excess Hx (no/yes)	0.70 (0.13–3.79)	0.679	–	–
BMI (< 25/≥ 25 kg/m^2^)	0.77 (0.28–2.14)	0.612	–	–
MUST (low/medium–high risk)	2.04 (0.60–6.89)	0.253	–	–
Serum CRP (≤ 10/11–80/> 80 mg/L)	1.40 (0.77–2.52)	0.268	–	–
Oxygen requirement (no/yes)	1.68 (0.73–3.89)	0.224	–	–
ITU admission (no/yes)	0.91 (0.25–3.25)	0.883	–	–
30-day mortality (no/yes)	1.03 (0.28–3.76)	0.970	–	–

## Discussion

To our knowledge, the present study is the first to examine the relationships between micronutrient status, frailty, systemic inflammation and clinical outcomes in patients admitted with COVID-19. The results of the present study show that in the non-frail patients (younger, healthier and less malnourished), over a third of patients (36%) had low vitamin B1. This was in contrast to the other red cell measures including B2, B6 and selenium in which no patients were deficient (see Table 3). Furthermore, that median vitamin B1 concentration was inversely related to the magnitude of systemic inflammatory response (SIR). Therefore, although the basis of the deficiency is unclear, targeted supplementation of thiamine may have the potential to improve clinical outcomes in patients with COVID-19. Particularly, in those patients who experience a significant SIR, a robust prognostic factor adversely associated with clinical outcomes in COVID-19 [32, 33] and a risk factor for developing long COVID [34, 35].

Postulated as a therapeutic target in patients with COVID-19 [23], the prevalence of thiamine deficiency in COVID-19 patients remains unknown. Indeed, to date, preliminary studies examining the effects of thiamine supplementation on clinical outcomes in patients with COVID-19 have failed to report baseline thiamine status or the relationship with other red cell vitamins [25]. Therefore, the present observations are informative finding that on a background of normal reference range values of the other red cell vitamins, over a third (36%) of non-frail patients admitted with COVID-19 had a low vitamin B1 (thiamine). Given the prevalence of vitamin B1 deficiency in the present study is higher than that reported by contemporary studies of critically unwell patients (20%) [36, 37], it provides rational for thiamine supplementation in patients admitted with COVID-19. However, comparative studies of healthy individuals are required to whether the vitamin B1 deficiencies observed are endemic to our population post-pandemic.

While a low vitamin B1 was not found to be significantly associated with SIR or clinical outcomes in the present study (see Table 5), median vitamin B1 concentration was found to inversely related to magnitude of SIR, a robust prognostic factor [32]. Indeed, the lowest median vitamin B1 concentration was observed in patients experiencing the highest magnitude of SIR (CRP > 80 mg/L). As such, the present observations suggest that vitamin B1 stores may be depleted in COVID-19 patients experiencing a higher magnitude of SIR. One hypothesis for the thiamine deficiency observed in patients with COVID-19 is increased consumption to meet the energy demands of protein and ribonucleotides synthesis for viral replication [37]. If confirmed in future studies, this may have a number of clinical implications. Firstly, that thiamine supplementation may be required to replenish depleted stores in hospitalized COVID-19 patients who experience a high magnitude of SIR [25, 38]. Secondly, given the similarities in symptoms of thiamine deficiency [39, 40], depletion of vitamin B1 stores may explain the basis of the relationship between a high magnitude of SIR during COVID-19 infection with the development of long COVID [34, 35]. Further studies of thiamine status following recovery from COVID-19 and in those with long COVID are therefore warranted.

Deficiencies in serum/plasma micronutrients, including vitamin D, selenium and copper have been associated with disease severity and poorer clinical outcomes in patients with COVID-19 [8, 9, 41]. However, these studies have often been carried out using measurements that are recognised to be perturbed by the SIR [13, 42, 43]. This was highlighted in a recent meta-analysis by Oscanoa and co-workers who stated that it was unclear whether the deficiencies observed in Vitamin D was specific to COVID-19 severity or simply a consequence of the cytokine storm typically exhibited in patients with severe disease [26]. Indeed, both plasma selenium and copper were found to be associated with magnitude of SIR in the present study, in keeping with contemporary literature [42, 44, 45]. Furthermore, despite nearly half of patients studied (45%) found to have low plasma selenium, no patients had a low red cell selenium-thought to be a reliable indicator not confounded by the SIR [21]. As such, the therapeutic benefit of supplementation cannot be determined as it remains unclear if there is a casual effect between micronutrient deficiencies (assessed by measurement of their concentration in plasma) and clinical outcomes in patients with COVID-19, or if these are solely reflective of the magnitude of the SIR [2].

The present study has a number of limitations. Firstly, given the relatively small sample size, the present study may be subject to sample bias. Secondly, there is also potential for selection bias with micronutrient screening conducted in only 47% (n = 281) of the 599 patients admitted to hospital with COVID-19 during the study time frame. However, the clinicopathological characteristics of the included patients were similar to that of patients in the overall cohort [46]. Therefore, this was not considered to be a significant confounding factor to the present observations. Thirdly, analysis of all eight micronutrients examined in the present study was not possible in all patients included due to limited blood sample availability. As such, examination of micronutrient perturbation specific to COVID-19 was limited. Lastly, the absence of follow-up micronutrient screening to examine trends in status is a limitation. Serial measurements would be useful to delineate whether the deficiencies in micronutrients are a result of COVID-19 per se, or simply that COVID-19 occurred on a background of micronutrient deficiency.

## Conclusion

In patients admitted with COVID-19 who were not frail, over a third (36%) of patients had low vitamin B1. Furthermore, median vitamin B1 concentration was inversely associated with a higher magnitude of SIR. Therefore, in patients experiencing a significant SIR, thiamine stores may be depleted and provide rationale for supplementation. Further longitudinal studies are warranted to delineate the trend in thiamine status following COVID-19.

## Data Availability

Data will be made available upon request and approval of senior author (DM).
